# Auricular reconstruction after Mohs micrographic surgery: analysis of 101 cases^☆^^☆☆^

**DOI:** 10.1016/j.abd.2020.12.008

**Published:** 2021-06-03

**Authors:** Guilherme Canho Bittner, Elisa Mayumi Kubo, Bruno de Carvalho Fantini, Felipe Bochnia Cerci

**Affiliations:** a Dermatology Service, Universidade Federal de Mato Grosso do Sul, Campo Grande, MS, Brazil; b Private Practice, Campo Grande, MS, Brazil; c Private Practice, Porto União, PR, Brazil; d Dermatology Service, Universidade de São Paulo, Hospital das Clínicas, Faculdade de Medicina de Ribeirão Preto, Ribeirão Preto, SP, Brazil; e Dermatology Service, Hospital de Clínicas, Universidade Federal do Paraná, Curitiba, PR, Brazil; f Postgraduate Program in Internal Medicine and Health Sciences, Universidade Federal do Paraná, Curitiba, PR, Brazil; g Private Practice, Curitiba, PR, Brazil

**Keywords:** Auricular pavilion, Ear neoplasms, Mohs surgery, Surgical flaps

## Abstract

**Background:**

The ear is a region that has a high prevalence of cutaneous carcinomas and several guidelines indicate Mohs micrographic surgery as the first-choice treatment in such cases. Although the technique allows maximum preservation of healthy tissue, many auricular surgical wounds constitute a challenge due to the peculiar local anatomy, with evident curves and reliefs. Auricular reconstruction should prioritize function before aesthetics, but without leaving the latter aside, since postoperative distortions can have a significant psychological impact.

**Objective:**

To describe the authors’ experience in auricular reconstruction after Mohs surgery and to evaluate the most frequently used repair methods.

**Methods:**

Retrospective study of consecutive cases submitted to Mohs surgery and auricular reconstruction, over a period of 3 years.

**Results:**

One hundred and one cases were included and the most common repair method was primary closure (n = 35), followed by full-thickness skin graft (n = 30) and flaps (n = 24). In thirty cases, reconstruction methods were associated. Seven patients had complications (partial graft necrosis, postoperative bleeding or infection).

**Study limitations:**

Retrospective design and the absence of long-term follow-up of some cases.

**Conclusions:**

The dermatologic surgeon should be familiarized with different options for auricular reconstruction. Primary closure and skin grafts were the most frequently used repair methods.

## Introduction

The ear is a region that has a high prevalence of cutaneous carcinomas.^1^ Due to its aesthetic and functional importance, it is essential to offer patients treatments with less chance of recurrence. For this reason, several guidelines indicate Mohs micrographic surgery (MMS) as the first-choice treatment for cutaneous carcinomas at this site.^2^, ^3^, ^4^ MMS has the highest cure rates in the treatment of *cutaneous* *carcinomas*, as it evaluates 100% of the surgical margins, whereas the wide local excision technique assesses about 1% of the margins by "bread-load" sectioning.^5^, ^6^

Although MMS allows maximum preservation of healthy tissue, many auricular surgical wounds are a challenge due to the peculiar local anatomy, with evident curves and reliefs. Auricular reconstruction should prioritize function before aesthetics, but without leaving the latter aside, since postoperative distortions can have a significant psychological impact.^7^ It is important to mention that the ear is essential for the support of prescription glasses and the “fitting” of hearing aids, especially in elderly patients.

For the repair of auricular surgical wounds, one must take into account their diameter and depth, availability of adjacent skin, cartilage involvement, use of hearing aids, dependence on prescription glasses, and patient expectations.^8^, ^9^ There are several available options, including healing by secondary intention, primary closure, skin grafts, cartilage grafts, flaps, or a combination of them.

The aim of the study was to describe the authors’ experience in auricular reconstruction after MMS and to evaluate the most frequently used repair methods.

## Methods

This was a retrospective study of consecutive cases submitted to MMS and auricular reconstruction by the authors, between January 2017 and July 2020. The cases are from the authors’ private practices and from a university hospital where one of the authors works. The study was approved by the Ethics Committee, protocol number 30746120.4.0000.0103.

With the exception of one surgery performed under local anesthesia and sedation, all were performed under local anesthesia. Postoperatively, antibiotics (cephalexin 500 mg, every 6 h for 7 days or cefadroxil 500 mg, every 12 h for 4 days) were used in more complex, long-term surgeries or when cartilage was removed.

For data analysis, a review of the photographic documentation and the following data were performed: age, sex, Fitzpatrick phototype, tumor characteristics, defect size and involved anatomical subunits, number of MMS stages, type of reconstruction performed, use of platelet antiaggregants or anticoagulants and postoperative complications.

**Figure 1:**
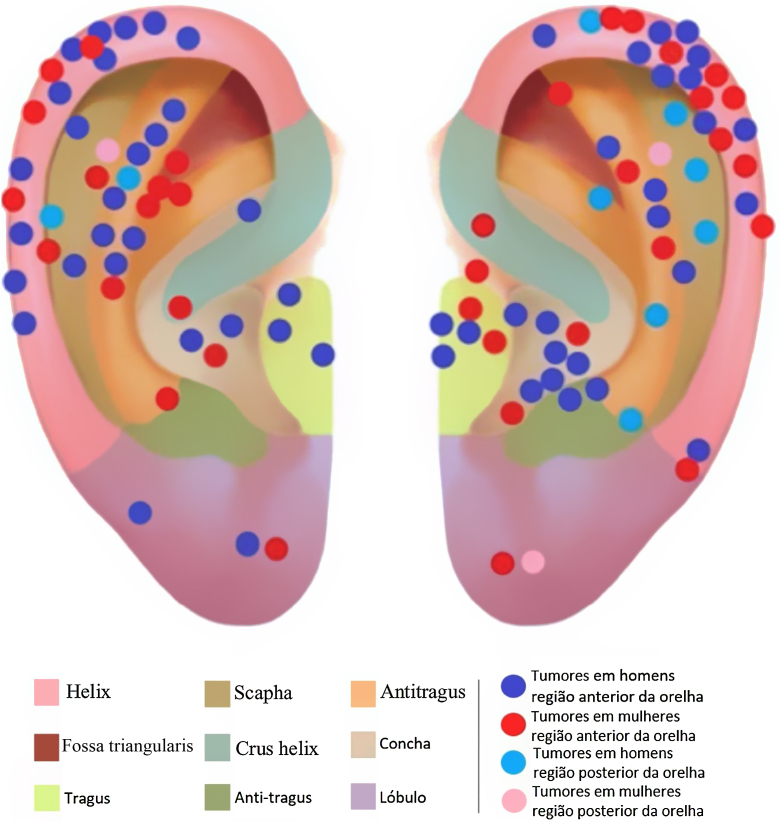
Tumor location. * The auditory canal and the tumor located in it are not shown in the picture.

The auricular subunits were divided into crus helix, helix, anti-helix, concha, tragus, antitragus, scapha, lobule, the auditory canal and posterior region of the ear (Fig. 1). The reconstruction methods were divided into healing by second intention, primary closure, flaps or graft. When more than one method was used, it was called a combined reconstruction. For the analysis of repair methods, only repairs used for the closure of auricular subunits were considered.

Complications were divided into two groups, short and long-term ones. The following were considered short-term: bleeding that required reintervention, hematoma, infection, dehiscence, and flap/graft necrosis (partial or total). Long-term ones were defined as considerable anatomical distortions and chondritis.

## Results

A total of 101 cases of 90 patients were included in the study. In 99 cases, the surgical wounds were reconstructed by the dermatologists themselves after the end of the MMS. Demographic and surgical data are described in Table 1. The mean size of the tumors was 11.7 × 9.4 mm, with 87 primary, 11 recurrent and 3 incompletely excised tumors. Fourteen patients were on acetylsalicylic acid, two on rivaroxaban, and one on clopidogrel.

**Table 1:** Demographic and surgical data of the 101 surgical cases.

Age (years)	Sex	Phototype	Reconstruction method^a^
68.9 (mean)	37 women	I:1	Primary closure: 35
44 to 92	53 men	II: 60	Skin graft: 30
		III: 29	Flap: 25
			Second intention: 11

Number of stages	Tumors	Anesthesia method	Mean defect size (mm)
1.4 (1 to 4)	84 BCCs	Local: 100	15.8 × 12.5
	10 SCCs	Local + IV sedation: 1	
	6 SCCs *in situ*		
	1 keratoacanthoma		

a In this division, only the main method of reconstruction was accounted for. In 33 cases, there was a combination of repair methods, with the most common combination being primary closure with skin graft (n = 14).

The primarily affected auricular subunits were the helix (n = 38), followed by the antihelix (17), concha (12), posterior ear surface (11), tragus (7), crus helix (5), lobule (5), fossa triangularis (2), scapha (2), antitragus (1) and the auditory canal (1). In 77 cases, only one subunit was affected and in 24 cases, more than one subunit was involved. The combination of reconstruction methods was used in 18.2% of cases with the involvement of one subunit and 62.5% of cases with the involvement of more than one subunit.

The most frequently used reconstruction methods were: primary closure (n = 35) and skin graft (n = 30). In four cases, cartilage grafting was performed concomitantly with the interpolation flap. Table 2 shows the reconstructions performed according to the involved auricular subunit. For the helix, tragus and posterior surface of the ear, the primary closure was the most frequently performed; for the antihelix, full-thickness skin graft; for the crus helix, flaps and, for the concha, healing by second intention.

**Table 2:** Reconstructions performed according to the affected subunits.

	Number of cases	Second intention	Primary closure	Flap	Graft
Helix	38	–	16	11	11
Antihelix	17	1	2	3	11
Concha	12	6	1	3	2
Posterior area	11	1	5	2	3
Tragus	7	–	6	1	–
Lobule	5	–	4	1	–
Crus helix	5	–	1	3	1
Scapha	2	1	–	–	1
Fossa triangularis	2	1	–	–	1
Antitragus	1	–	1	–	–
Auditory canal	1	1	–	–	–

Regarding the flaps, in 24 cases, they were the main reconstruction method: interpolation (n = 5), pull through (n = 5), transposition (n = 5), advancement (n = 4), rotation (n = 3) and island pedicle (n = 2). There was a combination of reconstruction methods in 30 cases. Two patients were referred by plastic surgery to undergo MMS and, after its completion, returned for reconstruction with the plastic surgeon. One was repaired using primary closure and the other with an advancement flap.

Pre- and postoperative prophylactic antibiotics were used in 12 and 44 cases, respectively. Complications occurred in 6.93% (n = 7) of the cases, all were short-term. The most common complications were partial graft necrosis (n = 5) and bleeding (n = 2), none was life-threatening. There was one case of local infection (associated with graft necrosis), which was treated with oral antibiotics with adequate resolution. Partial flap or graft necrosis were managed with local wound care, without significant long-term damage.

## Discussion

The present study demonstrated the variety of reconstruction options available for the auricular region, in addition to the frequent use of flaps and grafts, similarly to previous publications.^10^, ^11^ It also demonstrated that, differently from the nasal area, the auricular region allows the reconstruction of several anatomical subunits with the same method, either by a flap or graft, without aesthetic prejudice to the patient.

The mean number of stages for complete tumor resection was 1.4. A possible explanation for the relatively small number of stages was the dermoscopic examination in the preoperative period, associated with a high number of primary cases in the present study.

It is interesting to note that 10% of patients (n = 9) had concomitant tumors in the auricular region, representing 19.8% (n = 20) of all lesions. Despite the small number, the hypothesis that patients with cutaneous carcinomas in the ear are at greater risk of having a new lesion in the same anatomical region can be suggested. This number may be even higher if we take into account that other patients may seek treatment from other professionals.

The choice of repair methods varied according to the location and extent of the surgical defect, with primary closure being the most frequently used (n = 35). This reconstruction method showed to be valuable, mainly for small-extension lesions, and in 80% of primary closure cases, surgical defects had up to 13 mm in their largest extension. In the remaining 20%, the wounds were located in more mobile subunits, such as the lobule and posterior ear surface.

**Figure 2:**
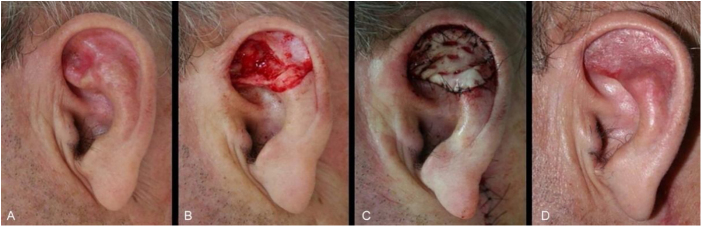
Full-thickness skin graft for multiple auricular subunits. (A), Poorly delimited basal cell carcinoma. (B), Surgical defect involving the scapha, anti-helix and fossa triangularis with loss of cartilage. (C), Immediate postoperative period. (D), Long-term postoperative.

The second most frequently repair was full-thickness skin graft (n = 30). This method was implemented in several subunits (helix, crus helix, scapha, antihelix, concha, fossa triangularis, and posterior ear surface), for wounds of variable sizes, however, that did not have significant loss of contour or cartilaginous structure of the ear. Defects with cartilage loss can be restored with a skin graft, ideally if the ear cartilaginous structural support is maintained, to preserve its contour and function (support for glasses, for example) (Fig. 2).

**Figure 3:**
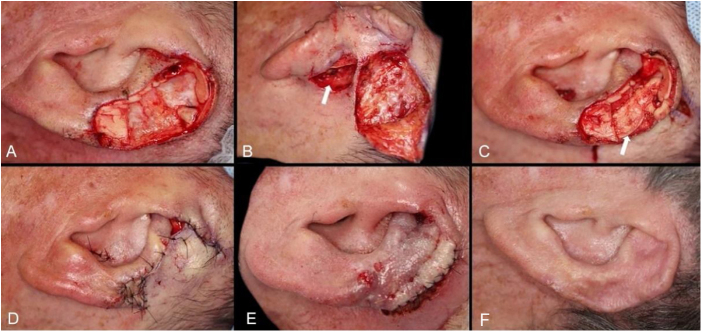
Interpolation flap from the mastoid area. (A), Surgical defect involving multiple subunits, with significant loss of cartilage. (B), Detached interpolation flap. The lower incision was performed to harvest the cartilage graft from the concha (white arrow). (C), Sutured cartilage graft (2.5 × 1 cm) (white arrow). (D), Immediate postoperative period. A small skin graft was used for the lower portion of the wound. A small area in the scaphoid fossa was allowed to heal by second intention. (E), Immediate postoperative period of the second surgery (pedicle division), performed 3 weeks later. (F), One-year postoperative period. Cartilage replacement was essential to prevent contraction of the ear.

**Figure 4:**
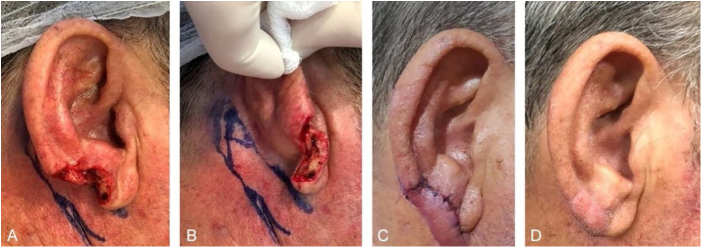
Retroauricular interpolation flap for the lobule. (A), Full-thickness surgical defect. (B), Flap design. (C), One week after the first stage. Note the pedicle at the bottom part of the figure. (D), Three months of postoperative period. Note the adequate repair of the lobule contour and maintenance of the ear size.

The interpolation flap from the mastoid area, a reconstruction option for extensive defects (mean size of 36.4 × 26.8 mm), was used mainly when there was concomitant involvement of the helix, scapha and antihelix (Fig. 3). In all cases, the wounds were full-thickness and, in 4 cases, they were associated with cartilage graft to maintain or restore the auricular contour. The cartilage grafts were obtained from the ipsilateral concha (Fig. 3). In one case, the interpolation flap was used to repair the lobule (Fig. 4). Despite the need for two surgeries for its completion, this flap allows repairing extensive auricular wounds, even full-thickness ones.^12^, ^13^

**Figure 5:**
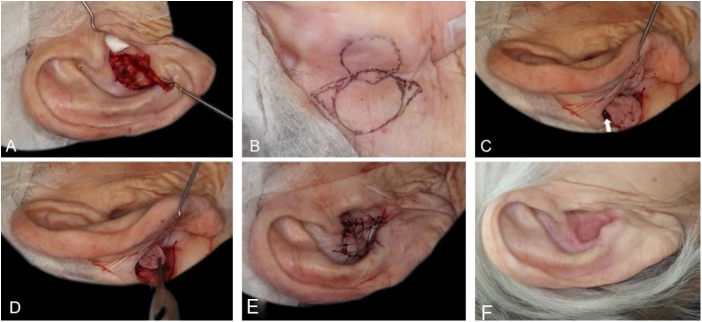
Pull-through flap. (A), Surgical defect involving the concha, with loss of cartilage. (B), Flap design. (C), Detached flap with island pedicle. The white arrow indicates the flap movement. (D), Flap crossing the ear after a small full-thickness incision in the concha. (E), Immediate postoperative period. (F), One year of postoperative period.

The pull-through flap was used mainly to repair the concha and antihelix subunits. Larger and deeper surgical wounds in these areas might not have the skin graft as the best indication, due to the increased risk of necrosis, especially if the perichondrium has been removed. In the pull-through flap, the skin of the mastoid region in the form of a “saloon door” flap is used for the complete closure of the wound (Fig. 5).^14^ This flap is performed in a single stage, therefore a new approach is not necessary after three weeks. Compared to second-intention healing, an excellent option for the concha, the advantage of this flap is the immediate repair of the wound, which shortens and minimizes postoperative care.

**Figure 6:**
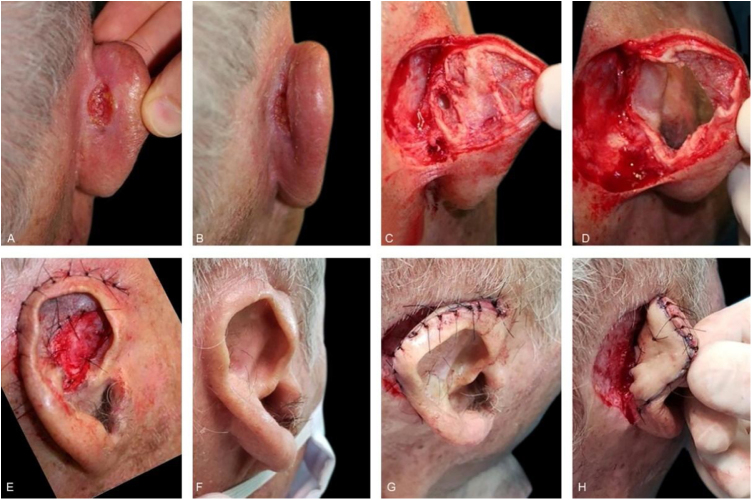
(A and B), Recurrent SCC in the retroauricular sulcus. (C), Surgical defect after the first stage of Mohs surgery. The deep margin was completely affected, even where the cartilage had been removed. (D), Final surgical defect involving the retroauricular area (retroauricular sulcus) and full-thickness of the pavilion (almost the entire anti-helix and fossa triangularis). The helix was preserved. (E), Immediate postoperative period. The pavilion was sutured in the retroauricular/mastoid region. The area of the full-thickness defect healed by second intention. (F), Postoperative. (G and H), After months, at a second moment, the pavilion was detached from the retroauricular region and a full-thickness skin graft (harvested from the supraclavicular region) was performed in the posterior surface of the pavilion. The retroauricular area healed by second intention.

Second-intention healing was chosen for defects in concave areas (concha, scapha, fossa triangularis, and auditory canal) and superficial wounds of the anti-helix and posterior region with preserved perichondrium. When the latter is involved, the healing takes longer, but it does not contraindicate healing by second intention.^15^ To optimize this time, 2-mm punches can be made, transfixing the cartilage and allowing the wound to be nourished from the opposite side. This technique can also be associated with skin grafts. Second-intention healing can be combined with other repair methods. In the present study, this occurred in nine cases, as shown in Fig. 6.

**Figure 7:**
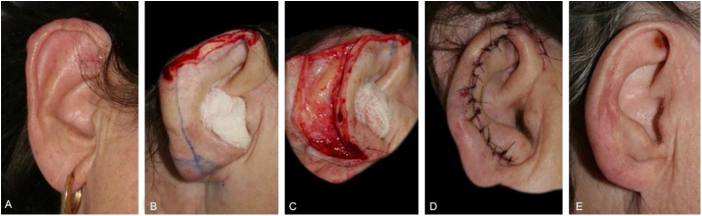
Helix advancement flap. (A), Poorly circumscribed basal cell carcinoma on the right helix. (B), Full-thickness surgical defect with loss of the helix contour. (C), Detached flap. (D), Immediate postoperative period. (E), Long-term postoperative.

**Figure 8:**
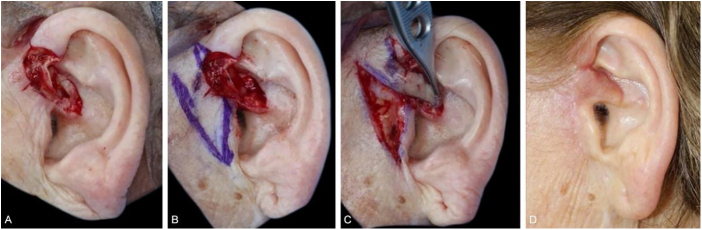
Pre-auricular transposition flap to the crus helix. (A), Surgical defect involving the crus helix with loss of cartilage. (B), Flap design. (C), Flap movement. (D), Eighteen months of postoperative (new image).

Traditional flaps such as transposition, advancement, rotation, and island pedicle (V – Y) were used on 14 wounds, all with dimensions >12 mm. The advancement and island advancement flaps were implemented mainly for the helix region, recruiting skin from the posterior and/or inferior portion of the ear. The use of the rotation flaps for full-thickness helical defects is an alternative to the interpolation flap (Fig. 7). The transposition flap also showed to be an excellent alternative for anterior portion defects, such as the crus of the antihelix and tragus, as it uses the skin of the pre-auricular region, resulting in a discreet scar in the donor area (Fig. 8).

The use of prophylactic antibiotics in the pre-and postoperative periods is a controversial topic in dermatologic surgery. Some authors recommend preoperative use when the auricular cartilage is involved, as well when using grafts and flaps on the nasal region.^16^ In the present study, preoperative antibiotic therapy was indicated in 11.8% and postoperative antibiotics in 43.5% of cases, reflecting the difficulty in anticipating the wound extent and the type of reconstruction to be performed.

The performance of the procedures with local anesthesia and vasoconstrictors (100 cases) in the outpatient setting (95 cases) did not show an increase in risks or complications for the patients, data that have been well established in the literature.^17^, ^18^, ^19^, ^20^, ^21^ With 0.99% of infection and 6.93% of minor complications (bleeding and partial graft necrosis), the authors’ sample reproduces the types of adversities most often described in the literature.^10^, ^19^

## Conclusion

Although primary closure and grafts correspond to the main methods of auricular repair, the dermatologic surgeon must become familiar with several reconstruction alternatives in this area, aiming at functional preservation and aesthetic restoration. It is essential to reinforce that, as recommended by the National Comprehensive Cancer Network, ideally, reconstructions in cosmetic sensitive areas such as the ear are performed after complete tumor resection, confirmed by analysis of 100% of the surgical margins during surgery, as performed in MMS.^3^

## Financial support

None declared.

## Authors’ contributions

Guilherme Canho Bittner: Participation in the design and planning of the study; collection, analysis, and interpretation of data; writing; approval of the final version of the manuscript.

Elisa Mayumi Kubo: Writing; approval of the final version of the manuscript.

Bruno de Carvalho Fantini: Collection, analysis, and interpretation of data; critical review of the manuscript; approval of the final version of the manuscript.

Felipe Bochnia Cerci: Participation in the design and planning of the study; collection, analysis, and interpretation of data; writing; critical review of the manuscript; approval of the final version of the manuscript.

## Conflicts of interest

None declared.
